# Satisfaction With Children’s Achievements and Health Outcomes in a Sample of Community Older Adults in Nigeria

**DOI:** 10.1093/geroni/igad088

**Published:** 2023-08-22

**Authors:** Babatola Dominic Olawa

**Affiliations:** Institute for Gerontology, University of Vechta, Vechta, Lower Saxony, Germany; Department of Psychology, Federal University Oye-Ekiti, Oye-Ekiti, Ekiti State, Nigeria

**Keywords:** Anxiety, Children’s achievements, Depression, Self-rated health, Somatic health

## Abstract

**Background and Objectives:**

In most African societies with little or no social welfare services for older adults, many parents regard their children as personal investments and security for the future. As a result, satisfaction with children’s achievements may be necessary for older parents’ physical and mental health. This study examined the association between satisfaction with adult children’s achievements (SACA) and health outcomes (regarding somatic health, self-rated health, anxiety, and depression) in a sample of Nigerian older adults.

**Research Design and Methods:**

By using the multistage sampling technique in a cross-sectional survey, 465 older adults (mean age = 74.18 ± 9.42) consisting of 294 women were selected from 14 rural communities in a State in Nigeria. Data were collected using validated instruments and analyzed using multiple linear regression and multigroup analyses in SPSS AMOS.

**Results:**

High SACA was significantly associated with low somatic health problems, positive self-rated health, and low levels of anxiety and depression, even after controlling for sociodemographic factors, children’s support, social engagement, and loneliness. Further analyses indicated that the relationship between SACA and depression was only significant in women and more robust in the widowed. Also, SACA was associated with somatic health among the married but not the widowed. The level of children’s support was not a significant moderator.

**Discussion and Implications:**

Parents can experience positive emotions and, consequently, good health from being satisfied with their children’s achievements regardless of perceived children’s support. Assessing SACA may aid in better diagnoses and formulation of a more effective clinical intervention to improve the well-being of older adults in developing societies.

**Translational Significance:** Given that satisfaction with adult children’s achievements (SACA) is associated with somatic health, self-rated health, anxiety, and depression in this study, clinicians need to assess for SACA during evaluation as appropriate to ascertain its contributions to mental and somatic health problems among older adults. The outcome of such assessment can necessitate techniques such as cognitive behavioral therapy and gratitude interventions to boost the level of SACA for improved geriatric mental and somatic health.

The global population of older adults is projected to rise from 727 million in 2020 to 1.5 billion by 2050 due to the increase in life expectancy and decrease in birth rates (United Nations, 2020). Aging population by region shows that only 5.6% of Africans are 60 years and older, an aging population proportionally less than Asia (13.3%), Europe (25.5%), Latin America, and the Caribbean (12.8%), Northern America (23.4%), and Oceania (17.9%; He, 2022). By the year 2050, however, the share of older adults in Africa will exceed that of America, nearly equal to Europe, with Nigeria predicted to have the largest share in the region (He, 2022). To meet this projection, African policymakers must begin to plan for appropriate holistic geriatric care as old-age dependency rises. As chronological age increases, gradual and progressive biological aging is accompanied by a deterioration in system integrity and subsequent frailty and ill health (Belsky et al., 2020).

Over the past two decades, the rise in the burden of noncommunicable diseases (NCDs) in sub-Saharan Africa has posed serious problems for the health systems (Gouda et al., 2019). Specifically, NCDs such as diabetes, heart diseases, and cancer remain the primary causes of mortality among African older adults (World Health Organization, 2023). In addition to physical health burdens, African older adults are highly vulnerable to mental health distress from lack of food, health insecurities, poverty, and poor social protection (Adamek et al., 2022; Gyasi et al., 2020). Studies carried out across regions in Nigeria show that common mental disorders such as depression and anxiety are relatively prevalent among older people (Awunor et al., 2018; Gascoyne et al., 2022; Igbokwe et al., 2020; Ojeahere et al., 2022).

Adult children form part of the support and care network for older adults worldwide (Haberkern & Szydlik, 2010; Horioka et al., 2017). However, unlike in developed economies where formal care is provided, most older adults in Nigeria depend almost entirely on their adult children to meet social, economic, and health needs (Akinrolie et al., 2020; Mbam et al., 2022; Togonu-Bickersteth, 1989). More than 95% of Nigerian older adults do not have access to pension funds to meet their socioeconomic needs because they were not employed in the formal private or public sector during their active years (Dataphyte, 2019; Mbam et al., 2022). Hence, older adults must depend entirely on their children and other family members for financial sustenance. Unfortunately, given the poor socioeconomic conditions in Nigeria, many adult children cannot render adequate social and financial support to their older parents (Togonu-Bickersteth & Akinyemi, 2014). This reality implies that older parents’ well-being largely depends on the levels of adult children’s achievements.

Previous works already show that levels of children’s success in life spheres predict older adults’ health. For example, Zhang and Liu (2022) demonstrated that high levels of achievement of children in education and income predict greater positive mental well-being, good self-rated health, and physical health among Chinese older adults, with children’s financial support as a significant mediator. Similarly, Wang et al. (2022) documented that increased children’s achievements in occupation, finance, and education were associated with low depressive symptoms among older parents. On the contrary, children with low life achievements in relationships and careers are shown to evoke anger, disappointments, worry, guilt, and low fulfillment in parents (Cichy et al., 2013). Further, Chung and Park (2008) found the achievements of adult children as one of the three leading indicators of successful aging in addition to older people having good relationships with others and maintaining a positive attitude toward life.

The relationship between children’s achievements and older parents’ well-being is supported by the broaden-and-build theory, which suggests that the occurrence of positive emotions momentarily broadens collections of individuals’ thoughts and actions, which in turn function to build lasting personal resources spanning from intellectual and physical resources to psychological and social resources (Fredrickson, 2001). When children are high achievers, older parents experience positive emotions, engage in positive thoughts and actions, and experience an expansion of their coping resources, which further translates into positive health outcomes. Also, given that the “family investments” in children have paid off, parents would be happy, satisfied, and feel more positive emotions than negative ones. Based on the social capital theory, high levels of children’s success will boost the social resources of parents and consequently affect positive well-being. According to Lee (2018), children with more outstanding life achievements increase their parents’ social capital by offering more instrumental support, reducing parental burdens, and facilitating health-promoting behaviors for their parents.

## The Present Study

Though previous works have established the roles of adult children’s achievements on older parents’ well-being, the current study’s uniqueness is in assessing the relationship between satisfaction with adult children’s achievements (SACA) and health outcomes among older adults. The focus on satisfaction with children’s achievements rather than actual children’s success is premised on the proposition that parents see themselves in their children, perceive children’s success as part of their achievements, and utilize personal expectations to assess children’s level of achievements (Chung & Park, 2008; Levitzki, 2009; Ryff et al., 1994). As such, being satisfied with children’s achievements based on personal assessments may be equally as crucial as actual achievements. For example, children from two families may have similar achievements. However, their parents may express varied satisfaction levels with these achievements while considering differences in their expectations and past investments in the child. Because parents do not view children’s success in isolation but assess it based on their personal expectations, achievements, and aspirations (Pinquart & Ebeling, 2020; Ryff et al., 1994), this study extends previous knowledge by examining how being satisfied with the levels of children’s achievements associates with the health of older adults.

Moreover, research on the association between children’s success and the health of older adults is rare within the African context, where most older parents depend on their children for sustenance and support. One existing study in this area suggests that satisfaction with adult children success (SACA) relates to low geriatric depression in a sample of older adults (Olawa & Idemudia, 2019a). However, the study is limited to a single mental health outcome—depression, and did not examine whether the relationship between SACA and depression is moderated by levels of children’s support and sociodemographic characteristics such as gender, age, marital status, educational level, and employment status.

Examining the moderating roles of sociodemographic factors is essential, given, for example, that the beneficial effects of SACA on health may be more robust for women as they are more likely to have low economic power (Antczak & Zaidi, 2016; Ferrant et al., 2014) and thus receive more assistance from children than men. Given their more dependent conditions, the very old (i.e., 80+ years) and the widowed may require more children’s support than the young-old (i.e., <80 years) and the married, respectively (Lee et al., 2018; Olawa et al., 2021). Therefore, experience the more beneficial effects of SACA. Moreover, highly educated parents have greater expectations regarding their children’s achievements than less educated parents (Davis-Kean, 2005), which may affect the level of SACA and its relationship with health. For instance, parents with high expectations may experience lower health benefits from SACA than those with low expectations. Also, parents who are not gainfully employed may derive more health benefits from SACA than their counterparts who engage in paid employment because of the possibility of getting more children’s financial assistance. In addition, the role of SACA on health may be more substantial for parents who perceive more children’s support than those with a lower perception of children’s support.

Accordingly, this study contributes to the literature by examining the association between satisfaction with adult children’s achievement and physical and mental health measured by somatic health, self-rated health, geriatric anxiety, and depression. It further evaluates whether these relationships differ by sociodemographic factors and levels of children’s support. It is hypothesized that high SACA would relate to low somatic health problems, positive self-rated health, and decreased anxiety and depressive symptoms among older adults. It is also hypothesized that these relationships would differ by the levels of children’s support and sociodemographic factors of gender, age, marital status, educational level, and employment status. Understanding the role of SACA in health might be more practical for clinical interventions than actual children’s achievements, given that psychotherapy may be tailored towards increasing parental satisfaction with the levels of children’s achievements.

## Method

### Sample and Procedure

A sample of 465 older adults was selected using a multistage sampling technique from 14 communities in Ekiti State, southwest Nigeria. The Nigerian southwest comprises predominantly the Yoruba ethnic group. The Ekiti State was selected based on convenience and homogenous characteristics of the population, thus providing clear generalizability of findings (Jager et al., 2017). The sample comprises 294 women and 171 men, with an age range of 60 and 96 years and a mean age of 74.18 (*SD* = 9.42). The gender difference in sample size is consistent with the empirical findings that there are substantially more older women than men in all countries (United Nations, 2020). The majority of the participants were young-old [60–79 years: *n* = 301 (65%)], from polygynous families [*n* = 259 (56%)], informally employed [*n* = 265 (57%)], and had only primary level education [*n* = 355 (76%)]. Based on marital status, the number of widowed [*n* = 236 (50.8%)] was almost equal to the number of married [*n* = 229 (49.2%)].

The multistage sampling process involved randomly selecting 6 local government areas (LGAs) out of the 16 LGAs in the State. In the next stage, three communities were randomly chosen from the selected LGAs, except for two LGAs consisting of only one community each. The study settings were 14 communities in all. There was no formal registration of older adults in Nigeria, so a quasi-systematic random technique was utilized to select participants from their homes. Home-to-home contacts were made in streets and houses with odd numbers to achieve widespread coverage of each community selected. To be included in the study, participants must be ≥60 years old, able to communicate using the local dialect and/or English, and not have cognitive disorientation as confirmed by family members and during initial rapport preceding instruments’ administration. The Institutional Review Board approved the study’s ethical protocol, which follows the 2013 revised Helsinki Declaration.

### Instruments

#### Independent variable

Parental SACA was assessed using the Satisfaction with Adult Offspring’s Achievement Questionnaire (SOAQ; Olawa & Idemudia, 2019b). Participants were asked to express their overall satisfaction with the achievements of their adult children in six spheres of life, including education, health, finance, closeness to God, occupation, and marital/relationship. Responses were rated on a 5-point Likert scale ranging from *very dissatisfied* (0) to *very satisfied* (4). Olawa and Idemudia (2019b) demonstrated that the SOAQ is reliable and valid in measuring parental SACA within the Nigerian context. A Cronbach alpha coefficient of .75 was obtained for the scale in this study. High scores represent greater levels of satisfaction with children’s achievements.

#### Dependent variables

Health was assessed along four variables: somatic health, self-rated health, anxiety, and depression.

*Somatic health* was measured by asking participants to report the presence of the eight common diseases associated with aging as diagnosed by the physician (Bøen et al., 2012; Korten et al., 1999): diabetes, angina, chronic lung disease, musculoskeletal ailments, stroke, cancer, cardiac infarction, and osteoporosis. Responses were dichotomized into *yes* (1) or *no* (0). All *yes* responses were added together to derive a total score for somatic health (Bøen et al., 2012; Korten et al., 1999). *Self-rated health* was assessed with a single item by requesting participants to rate the overall condition of their health on a four-Likert scale format ranging from *poor* (1) to *excellent* (4) (Korten et al., 1999). The single-item self-rated health measure is widely accepted as a reliable and valid assessment of subjective health, just as the longer versions (Bowling, 2005; Cullati et al., 2020). *Anxiety* was measured using the Geriatric Anxiety Inventory—Short Form (GAI-SF; Byrne & Pachana, 2011). The GAI-SF consists of five items scored on a *disagree* (0) or *agree* (1) response format. The assessment of depression was carried out using the five-item Geriatric Depression Scale (GDS) and evaluated on a *Yes* (1) or *No* (0) response format. The reliability and the validity of the GAI-SF and the five-item GDS have been demonstrated previously within the Nigerian context (Olawa et al., 2020). During pilot testing, a 2-week test-retest of .85 and .90 was obtained for the GAI-SF and GDS. High scores on health indicators represent poor somatic health, good self-rated health, and high levels of depression and anxiety.

*Control/moderating variables.*— In addition to sociodemographics of gender, age, marital status, educational level, and employment status, children’s support, social engagement, and loneliness were controlled in the regression model, and these variables, except social engagement and loneliness, were then utilized as moderators in separate models. Children’s support was assessed with one item from the social support subscale of the Duke Social Support and Stress scale (Parkerson et al., 1989), asking participants to rate how supportive their children or grandchildren were. The response format was on a 3-point scale, *none* (0), *some* (1), and *a lot* (2). The Three-item Loneliness scale (Hughes et al., 2004) assessed loneliness on a 3-point response format, *hardly ever* (1), *some of the time* (2), and *often* (3). Social engagement was assessed by adapting the Mendes de Leon et al. (2003) social engagement measure, and rated on a 3-point scale, *never* (0), *sometime*s (1), and *often* (2). The list of social activities includes game playing, paid and unpaid community work, visits to family members, attendance at traditional ceremonies, going out to drink with friends, and participation in grassroots political activities. The psychometric soundness of the measures of loneliness, social engagement, and child support has been evident in the Nigerian context (Olawa & Idemudia, 2019a, 2020). All questionnaires were *transadapted* from English to Yoruba using the procedure of the International Test Commission (2005).

### Statistical Analyses

Multiple regression and multigroup analyses were calculated in SPSS AMOS. In a single model using the maximum likelihood method, somatic health problems, self-rated, depression, and anxiety were regressed on SACA while controlling for gender, age, marital status, occupation, education, children’s support, social engagement, and loneliness. Following the regression analysis, multigroup analyses were conducted to examine whether the relationship between SACA and health outcomes differed according to sociodemographics and levels of children support. Multigroup analysis was done by calculating the chi-square difference between the constrained and the model at *p* < .05 along the paths from SACA to the dependent variables. Given that children’s support is an ordinal variable, it was mean-centered, and an interaction term was generated with the SOAQ mean-centered scores for use in moderation analysis. Preanalyses data screening showed that data distribution was moderately normal, given that kurtosis and skewness scores were not above the cutoffs of 5 and 3, respectively (Byrne, 2010; Kline, 2011). A minimum sample size of 165 is required for a multiple regression model with 11 predictors with a statistical power of .80 and a moderate effect size of .15 at a *p* value of .05 (Soper, 2023). Hence, the sample size of this study (*N* = 465) is adequate to estimate the regression model.

## Results

### Association Between Satisfaction With Adult Children’s Achievement and Health Outcomes

Table 1 shows the results of the multiple linear regression examining the relationship between SACA and health outcomes. SACA was significantly associated with the somatic health (β = −.12, *p* = .012), self-rated health (β = .18, *p* < .001), anxiety (β = −.21, *p* < .001), and depression (β = −.19, *p* < .001). Specifically, high levels of SACA were associated with low somatic symptoms, positive self-rated health, low anxiety, and depression. Of all the control variables, gender, age, and loneliness were significantly associated with some aspects of health. The female gender was associated with more somatic symptoms (β = .16, *p* = .004) and high anxiety (β = .15, *p* = .01). Increasing age was related to poor somatic health (β = .17, *p* = .001) and poor self-rated health (β = −.11, *p* = .04). High loneliness was associated with poor somatic health (β = .10, *p* = .04), high anxiety (β = .27, *p* < .001), and depressive symptoms (β = .24, *p* < .001). Children’s support and social engagements were not significantly associated with health outcomes in the model.

**Table 1.** Association Between SACA and Health Outcomes Controlling for Other Variables

Variables	Somatic health			Self-rated health			Anxiety			Depression		
	*B*	*SE B*	β	*B*	*SE B*	β	*B*	*SE B*	β	*B*	*SE B*	β
SACA	−.04	.01	−.12^*^	.03	.01	.18^**^	−.09	.02	−.21^**^	−.04	.01	−.19^**^
*Control variables*												
Gender	.34	.15	.16^*^	−.11	.08	−.07	.53	.18	.15^*^	−.03	.10	−.02
Age	.02	.01	.17^**^	−.01	.004	−.11^*^	.004	.01	.02	.01	.01	.05
Marital status	.07	.15	.03	−.05	.08	−.04	.20	.18	.06	−.05	.10	−.03
Education	.01	.14	.003	−.17	.08	−.10^*^	.27	.17	.07	−.09	.10	.04
Employment	−.02	.13	−.01	.07	.07	.05	.02	.16	.01	−.02	.09	−.01
Children’s support	.07	.10	.04	.04	.06	.04	−.05	.07	−.03	−.07	.13	−.03
Social engagement	−.03	.02	−.06	.04	.01	.14	−.01	.03	−.02	−.02	.02	−.07
Loneliness	.07	.04	.10^*^	−.03	.02	−.07	.26	.05	.27^**^	.14	.03	.24^**^

*Notes*: SACA = Satisfaction with adult children’s achievements; *SE B* = standard error of the unstandardized beta weight. Gender (male = 0, female = 1); marital status (widowed = 0, married = 1); education (others = 0, primary = 1); employment (unemployed = 0; employed = 1).

^*^ *p* < .01.

^**^ *p* < .001.

### Results of Multigroup Analysis

Table 2 presents the moderating effect of sociodemographic variables on the association between SACA and health outcomes. Results indicated that gender moderated the relationship between SACA and depression [χ^2^ (1) = 10.20, *p* = .001]. The negative association between SACA and depression was found to be significant and stronger in women (β = −.35, *p* < .001) but insignificant among men (β = .04, *p* = .57). In addition, marital status significantly moderated the relationship of SACA with somatic health [χ^2^ (1) = 6.37, *p* = .01] and depression [χ^2^ (1) = 4.28, *p* = .039]. Specifically, the relationship between SACA and somatic health problems was significant among the married (β = −.27, *p* < .001) but not among the widowed (β = .003, *p* = .97). However, the association between SACA and depressive symptoms was stronger among widows (β = −.33, *p* < .001) than those married (β = −.16, *p* = .014). The moderating effects of age [χ^2^ (4) = 2.07, *p* = .72], education [χ^2^ (4) = 3.42, *p* = .49], and employment status [χ^2^ (4) = 7.78, *p* = .10] were not confirmed.

**Table 2.** Multigroup Moderation by Sociodemographics

Variables	Somatic health	Self-rated health	Anxiety	Depression	Unconstrained model	Constrained Model	∆χ^2^
	β	β	β	β	χ^2^ (df)	χ^2^ (df)	
*Gender*					217.18	229.96	12.77^*^
Male	−.23^**^	.20^**^	−.28^**^	.04			
Female	−.09	.23^**^	−.23	−.35^**^			
*Age*					217.71	219.14	1.43
Young-old	−.19^**^	.23^**^	−.23^**^	−.28^**^			
Oldest old	−.11	.24^**^	−.26^**^	−.20^**^			
*Marital status*					207.32	221.60	14.28^**^
Widowed	.003	.19^**^	−.27^**^	−.33^**^			
Married	−.23^**^	.25^**^	−.22^**^	−.16^*^			
*Education*					221.55	225.05	3.42
Primary	−.11^*^	.20^**^	−.19^**^	−.25^**^			
Others	−.13	.26^**^	−.42^**^	−.22^*^			
*Employment status*					221.02	228.79	7.78
Unemployed	−.19^**^	.18^**^	−.33^**^	−.16^**^			
Employed	−.09	.24^**^	−.17^**^	−.31^**^			

^*^ *p* < .01.

^**^ *p* < .001.

### The Moderating Role of Children’s Support

Table 3 showed that children’s support did not significantly moderate the relationship between SACA and health outcomes. The interaction term between SACA and children’s support was not significant on somatic health (β = −.001, *p* = .45), self-rated health (β = .06, *p* = .23), anxiety (β = −.04, *p* = .42), and depression (β = −.05, *p* = .32). However, children’s support was significant on self-rated health (β = .11, *p* = .04) and depression (β = −.10, *p* = .04) albeit at weak levels.

**Table 3.** The Moderating Effect of Children’s Support

Variables	Somatic health			Self-rated health			Anxiety			Depression		
	*B*	*SE B*	β	*B*	*SE B*	β	*B*	*SE B*	β	*B*	*SE B*	β
SACA	−.03	.02	−.11^*^	.04	.01	.19^**^	−.09	.02	−.23^**^	−.05	.01	−.21^**^
Children’s support (CS)	−.002	.10	−.001	.13	.06	.11^*^	−.17	.14	−.06	−.16	.08	−.10^*^
SACA × CS	.02	.02	.04	.02	.01	.06	−.02	.03	−.04	−.02	.02	−.05

*Notes*: SACA = Satisfaction with adult children’s achievements; *SE B* = standard error of the unstandardized beta weight.

^*^ *p* < .01.

^**^ *p* < .001.

## Discussion

Based on the established association between the life achievements of children and the health of older adults (Cichy et al., 2013; Wang et al., 2022; Zhang & Liu, 2022), the current study examined how parental satisfaction with these achievements relates to somatic health, self-rated health, depression, and anxiety and how sociodemographic factors and levels of children moderate these relationships support.

As hypothesized, the outcomes of multiple regression analysis showed that parental satisfaction with adult children’s achievement was significantly associated with health outcomes. High SACA was related to lower somatic health problems, positive self-rated health, low anxiety, and depressive symptoms even after controlling for sociodemographics, children’s support, social engagement, and loneliness. The results imply that older parents’ satisfaction with their children’s achievements can positively influence their well-being. Based on the tenets of the broaden-and-build theory (Fredrickson, 2001), satisfaction with adult children’s success can boost the positive emotions of parents in terms of happiness and fulfillment, which further promotes physical and psychological resources for positive physical and mental health. Satisfying children’s achievements can increase parents’ perception of children’s support and social capital base for promoting good well-being (Lee, 2018; Olawa & Idemudia, 2019a; Zhang & Liu, 2022).

In addition to the fact that African parents invest in children’s education so that they would become socially and economically independent when they become adults, parents also consider children as assets for their future and perceive children as their primary source of care and support when they reach the twilight years. This notion is entrenched, for example, in the Yoruba proverb that says, “Ti okete ba dagba, omu omo re lo nmu,” meaning “When the bush rat gets old, it feeds on its children’s breast” (Togonu-Bickersteth & Akinyemi, 2014, p. 365). Similarly, a Ghanaian proverb asserts that when your parents “take care of you when you cut your teeth, you must, in turn, take care of them while they are losing theirs” (Apt, 1996). Hence, achieving children are more likely to provide support and satisfaction for their older parents, which further protects against depressive symptoms (Olawa & Idemudia, 2019a; Wang et al., 2022), promotes positive self-rated and somatic health, and low anxiety feelings.

The outcomes of moderation analyses indicated that gender and marital status played significant moderating roles in the associations of SACA with depression and somatic health. Specifically, the protective role of SACA on depressive symptoms was observed among older women and not in older men. This confirms the study hypothesis that the association between SACA and health would be more robust in women than men. The gender difference may be accounted for by the variations in the levels of social and emotional connections of children with their parents during the advanced years. Compared to fathers, children tend to form deeper emotional relationships with their mothers (Lewis & Lamb, 2003; Portu-Zapirain, 2013; Zimmermann et al., 2022). These close ties put mothers in a better position to benefit more from children’s assistance. The possibility of women perceiving more support from their children may boost the negative association between SACA and depressive symptoms. Also, the low economic power of older women in most societies may position mothers to receive more assistance from their children (Antczak & Zaidi, 2016; Ferrant et al., 2014) and benefit more from the positive health consequences of children’s achievements. Independent of perceived children’s support, mothers may also feel more positive emotions from SACA based on their close attachments with their offspring and more profound identification with offspring achievements.

Although the association between SACA and depression was significant according to marital status, findings additionally show that this association was stronger among the widowed than the married. This line of result is anticipated because children occupy a central role in the lives of the widowed and tend to fill the gap created by spousal loss by providing much-needed support (Ha et al., 2006; Yang et al., 2022). As such, widows/widowers, compared to the married, are likely to experience more emotional benefits from SACA because contentment with their children’s success becomes one of the primary sources of consolation after spousal demise. In contrast, the direction of the role of SACA on somatic health was beneficial to the married compared to the widowed. SACA was significant on low somatic symptoms only among married individuals. This implies that SACA in itself may not produce health benefits but with interaction with the married state. In addition to the positivity from SACA, married older adults enjoy “marriage protection” (e.g., spousal physical and emotional support, spousal provision of health care and health information support), which may enable them to experience better physical health than those in the widowhood state (Pandey et al., 2019). The moderating roles of age, education, and employment status were not confirmed, implying that the roles of SACA on health outcomes along these factors are relatively invariant, disproving the study hypotheses.

However, one surprising finding is that levels of children’s support did not moderate the association between SACA and health outcomes. The relationship between SACA and health outcomes was anticipated to be stronger among older adults who report high levels of children’s support than those with less support. The result suggests that the positive benefits of SACA on older parents’ well-being may be independent of perceived support from children. This outcome provides credence to the broaden-and-build theory (Fredrickson, 2001) in that the protective role of SACA on health is fostered by the feelings of positive emotions associated with children’s achievements rather than perceived support from children. Although children’s support partly explains the mechanism by which children’s achievements and SACA affect good health outcomes (Lee, 2018; Olawa & Idemudia, 2019a; Zhang & Liu, 2022), parents can also derive positive emotions and, consequently, good health from their children’s achievements independent of whether the children support them or not. This is because parents psychologically “own” the achievements of their children as “Children’s success is parents’ success” (Yoo, 2006, p. 66).

### Limitations and Future Research Directions

There are some limitations to point out in this study. Given the study’s cross-sectional approach, the relationships between SACA and health outcomes are correlational and do not inform a cause–effect relationship. A longitudinal approach to data collection would enable tracking these relationships across time. Also, the study sample consists of Yoruba older adults living in rural communities in a southwestern State in Nigeria. This limitation does not permit the generalization of findings to older people in urban centers and States in other geopolitical zones of Nigeria that may have different sociocultural outlooks. Future studies in this area should consider a diverse sample comprising older adults from urban settings and other geopolitical zones. In addition, only parental satisfaction with the adult children’s achievements was assessed in the study. An additional evaluation of actual children’s achievements would have enabled the comparison of SACA with adult children’s achievements and identified which better accounts for variance in health outcomes among older adults. Further, the present study did not assess whether the gender of children has a role to play in the association between SACA and health outcomes, given the potential gender difference inherent in the level of children’s support to parents. Based on the assumption that parental expectations can influence the evaluation of children’s success, prospective studies may test whether SACA moderates or mediates the impact of children’s achievements (by gender) on physical and mental health in old age. In addition, the interaction effect between the socioeconomic status of parents and children’s achievements could be examined given that high children’s achievement coupled with high parental socioeconomic status could have a cumulative advantage on the health of older adults.

## Conclusion

This study confirms that satisfaction with adult children’s achievement is significantly associated with reduced somatic health problems, good self-rated health, and low anxiety and depressive symptoms in a sample of rural community older adults in Nigeria. However, some aspects of these relationships were moderated by gender and marital status. Perception of children’s support did not influence the association between SACA and health outcomes. Clinicians may need to assess the satisfaction levels with adult children’s achievements during evaluation to ascertain their contributions to mental and somatic health problems among older adults. The outcome of such assessment can necessitate techniques such as cognitive behavioral therapy and gratitude interventions to boost satisfaction with children’s success for improved geriatric mental and physical health.

## Data Availability

Data are made available upon request.
